# Vitamins and Vitamin-like Compounds in Severe Canine Periodontal Disease: A Prospective Exploratory Cross-Sectional Pilot Study

**DOI:** 10.3390/vetsci13090916

**Published:** 2026-09-07

**Authors:** Suzanna L. Hatunen, Jamie G. Anderson

**Affiliations:** 1 Veterinary Dental Services, Boxborough, MA 01719, USA; 2 School of Dental Medicine, University of Pennsylvania, Philadelphia, PA 19104, USA

**Keywords:** vitamins, micronutrients, nutrition, canine, dog, periodontal disease, oral health, one health

## Abstract

Vitamins and vitamin-like compounds are critical in human periodontitis pathogenesis. Limited reports on vitamin alterations in canine periodontal disease (PD) are available. A prospective, exploratory, cross-sectional pilot study evaluating plasma concentrations of vitamins and vitamin-like compounds by liquid chromatography and mass spectrometry was undertaken. The test cohort included 17 dogs with severe periodontitis, in at least two quadrants, and eight periodontally healthy control dogs seen for non-periodontal oral conditions. Strict inclusion and exclusion criteria were applied to the canine patients enrolled in this private specialty practice pilot study. After adjustment for multiple comparisons, only vitamin B9 (folate) was significantly associated with severe PD, with geometric mean concentrations approximately 28% lower in dogs with severe PD than in controls (0.00139 vs. 0.00193 μmol/L; adjusted *p* = 0.006). In exploratory unadjusted analyses, several additional metabolites had lower geometric mean concentrations in dogs with severe PD. Given the small and unbalanced sample and the inability to standardize diet, these findings should be interpreted as preliminary. Further investigation in larger, prospective, diet-controlled studies integrating vitamin profiling, metabolomics, and the oral microbiome is warranted.

## Simple Summary

Periodontal disease is common in dogs and is associated with inflammation and destruction of the tissues supporting the teeth. Vitamins are important for many biological processes, but their relationship with periodontal disease in dogs remains poorly understood. In this prospective, exploratory, cross-sectional pilot study, plasma concentrations of several vitamins and vitamin-related metabolites were compared between dogs with severe periodontal disease and dogs without periodontal disease. Dogs with severe periodontal disease had lower plasma folate (vitamin B9) concentrations than control dogs. Exploratory analyses also identified differences in several other vitamin-related metabolites; however, these findings were not significant after adjustment for multiple comparisons and require further investigation. The results demonstrate an association between lower plasma folate concentrations and severe periodontal disease but do not establish vitamin deficiency or a causal relationship. Larger studies with more balanced populations are needed to better understand the relationship between vitamin status and periodontal disease in dogs and to determine its biological and clinical significance.

## 1. Introduction

Periodontal disease (PD) is a chronic, progressive, inflammatory disease that when left untreated, can lead to destruction of periodontal tissue [1]. Severity, progression and an underlying predisposition for periodontitis appear multifactorial, involving a complex interaction between the environment, microbial community and host immune response [1,2]. In the human literature, it is well described that vitamins and nutritional status play important roles in maintaining periodontal health and in preventing PD [3,4,5,6,7]. Vitamins are essential nutrients that the body needs in small amounts to function optimally, playing vital roles in various physiological processes [5]. In people, deficiencies in several vitamins—including vitamin A (retinol), vitamin B1 (thiamin), vitamin B6 (pyridoxine), vitamin B9 (folate), vitamin B12 (cobalamin), vitamin C (ascorbic acid), vitamin D (calciferol), vitamin E (tocopherol) and vitamin K—have been associated with the presence or progression of periodontal disease compared to a healthy cohort [1,3,5,6,7,8,9,10,11,12]. The relationship between PD and micronutrients may be highly important given that it is a potential key modifiable factor in humans [12] and animals.

PD is one of the most commonly diagnosed conditions in dogs over three years of age [13]. Exploration of the potential role of vitamin levels in canine PD pathogenesis is limited. Only a few veterinary studies have focused on this potential causal factor for oral diseases. In one study [14], dogs with niacin deficiency had glossitis and stomatitis accompanied by ulceration. Deficiencies in niacin, riboflavin, pyridoxine, folic acid and pantothenic acid were also documented [15]. Pertaining specifically to PD, two recent vitamin studies are relevant. A 2024 article [16] revealed that the growth of *P. gulae* was significantly reduced after co-culture in vitro with 1% folic acid (FA) (*p* < 0.05), that generation of the halitosis components hydrogen sulfide and methyl mercaptan was reduced, and that pro-inflammatory cytokine production by *P. gulae*-infected human gingival epithelium was also reduced. Studies in the canine epithelium are needed to confirm the last finding. A more recent study in dogs showed that vitamin C and calcidiol (25-hydroxyvitamin D) levels were higher in periodontally healthier patients compared with patients with more severe periodontitis [17]. However, methodological differences in PD assessment should be considered when comparing other findings with the findings in our study.

Given the role that vitamins play in human periodontitis and the dearth of studies available in the canine literature, we hypothesized that dogs with severe periodontitis might demonstrate lower circulating plasma levels of key vitamins (A, Bs, C, D and E) and possible vitamin-like compounds. Therefore, this study aimed to quantify a broad panel of vitamins and vitamin analogs in canine patients with advanced PD compared to non-periodontitis healthy controls through liquid chromatography and mass spectrometry (LC-MS/MS). As applicable, the study also sought to compare vitamin-related associations between Stage IV periodontitis of dogs and those reported in human PD studies.

## 2. Materials and Methods

### 2.1. Study Design

This is a prospective, cross-sectional pilot study to quantify a broad panel of vitamins and vitamin-like compounds in canine patients with advanced PD and compare them to a healthy cohort.

### 2.2. Study Population

A total of 25 dogs were enrolled into the study from a private-practice dentistry and oral surgery referral hospital and evaluated by American Veterinary Dental College (AVDC) specialists. The study population included eight control dogs and 17 test dogs with severe PD. Selection of cases across multiple dog breed, size, sex and age groups was implemented to decrease any signalment bias in the results (refer to Table 1). Medical records for all 25 dogs were scrutinized to assess for eligibility and inclusion in the study.

### 2.3. Inclusion Criteria

Control cases were defined as dogs with Stage 0–1 PD across all quadrants. Test cases exhibited Stage 4 PD in two or more quadrants. Aligned with the AVDC definitions for periodontal disease staging (refer to Table 2) [18], Stage 0 (clinically normal) was defined as clinically healthy gum tissue with no gingivitis or attachment loss; Stage 1 PD (gingivitis) was defined as inflammation being present, but no loss of attachment; and Stage 4 PD (advanced periodontitis) was defined as greater than 50% attachment loss, with severe inflammation, infection, and significant tooth mobility being common. Control dogs were recruited from patients undergoing routine annual professional dental cleanings, malocclusion treatment, or treatment of focal traumatic endodontic disease (i.e., fractured tooth). Details of each patient’s clinical diagnosis and treatment are provided in Table 3.

#### 2.3.1. Periodontal Staging Criteria

All patients were required to have an awake oral exam, anesthetized oral exam and dental radiographs to assess and diagnose periodontal status.

- The awake examination served as a baseline for patient enrollment and subjectively evaluated gingival recession, gingivitis, exposed alveolar bone, furcation exposure, and missing or mobile teeth.
- The anesthetized oral exam assessed periodontal probing depth (millimeters), gingival recession (millimeters), gingival bleeding on probing (subjective scoring 0–3), tooth mobility (subjective scoring 0–3), evidence of furcation on probing (subjective scoring 0–3), tooth resorption, missing teeth, and/or any retained tooth material. If the status on anesthetized oral exam did not align with the inclusion criteria, the patient was not enrolled.
- Full-mouth dental radiographs were required in each patient to corroborate the periodontal staging of each patient.

#### 2.3.2. Patient Requirements

All patients required a complete blood count (CBC) and biochemistry blood panel, and were fasted prior to sample collection. In dogs older than seven years of age, a screening thyroid blood test (T4) was necessary. Dogs had to be on a commercially available complete and balanced diet that met AAFCO nutritional adequacy standards (either wet, dry or a combination). Patient data was recorded for analysis:

- Signalment and patient characteristics: patient age, breed, sex, neuter status and body weight (lb/kg).
- Pertinent medical history: concurrent systemic diseases, or other pertinent medical conditions.
- Current medications: long-term medications or supplements.

### 2.4. Exclusion Criteria

Dogs were excluded if they had received vitamin supplementation, selected non-vitamin nutritional supplements (fatty acids, prebiotics, probiotics, or postbiotics), or any systemic or topical antibacterial, antifungal, or immunosuppressive therapy within the previous three months. Additional exclusion criteria included a history of medications known to influence bone and mineral metabolism or periodontal health, including bisphosphonates, or long-term (>3 months) administration of corticosteroids, anticonvulsants, calcium channel blockers, or cyclosporine. Dogs with known renal or thyroid disease, concurrent generalized oral diseases (e.g., canine chronic ulcerative stomatitis, ulcerative mucosal disorders, oral masses, or suspected autoimmune oral disease), or suspected gastrointestinal malabsorptive disorders (e.g., chronic inflammatory enteropathy, exocrine pancreatic insufficiency, or chronic diarrhea) were also excluded. Dogs consuming a home-cooked diet, raw diet, or prescription diet were not eligible for enrollment.

### 2.5. Sample Collection

From each patient, 3 mL of fasted whole blood was collected into pre-chilled EDTA tubes by standard venipuncture and held on ice until centrifugation within 30 min at 4 °C. In total, 200 µL of plasma was aliquoted using a micropipette into amber Eppendorf tubes (Eppendorf SE, Hamburg, Germany) under low-light conditions. Plasma samples were immediately labeled, stored at −20 °C and shipped on dry ice to the laboratory for vitamin and vitamin-like compound quantification service analysis within 90 days of collection. All sample collection was performed in an identical manner by two technicians who followed a rigorous protocol. Sample labeling, storage and shipping were handled by the same board-certified dentist (SH) for consistency.

### 2.6. Laboratory Analysis

In the laboratory assays, linear-regression calibration curves of the analytes were constructed with the data acquired from the calibration solutions. Concentrations of detected compounds were calculated by interpolating the calibration curves with the data acquired from the sample solutions.

A total of 25 water-soluble vitamins and vitamin-like compounds were analyzed and quantified, including 4-aminobenzoic acid, lipoic acid, vitamin B1 (thiamine), vitamin B1 thiamine monophosphate (thiamine-P), vitamin B1 thiamine pyrophosphate (thiamine-PP), vitamin B2 (riboflavin), vitamin B2—flavin adenine dinucleotide (FAD), riboflavin-5′-phosphate (FMN), vitamin B3 (nicotinic acid), vitamin B3 (nicotinamide), vitamin B5 (pantothenate), vitamin B6 (pyridoxal), pyridoxal-5′-phosphate (PLP), pyridoxamine, pyridoxine, vitamin B7 (biotin), vitamin B9 (5-methyltetrahydrofolate), vitamin B9 (folate), vitamin C (ascorbic acid), vitamin B12 (cobalamin), adenosylcobalamin (ado-CBM), cyanocobalamin (cyano-CBM), methylcobalamin (methyl-CBM), hydroxocobalamin (OH-CBM), and total vitamin B12 (total B12).

A total of 21 fat-soluble vitamins and vitamin-like compounds were analyzed and quantified, including all-trans retinol, all-trans retinal, all-trans retinoic acid, retinyl acetate, retinyl palmitate, 1-hydroxyvitamin D2, vitamin D2, 1-hydroxyvitamin D3, vitamin D3, α-tocopherol, β-tocopherol, γ-tocopherol, α-tocotrienol, β-tocotrienol, γ-tocotrienol, tocopheryl acetate, vitamin K1, vitamin K2 (menaquinone-4; MK-4), vitamin K2 (menaquinone-7; MK-7), vitamin K2 (menaquinone-9; MK-9), and vitamin K3.

### 2.7. Ethics

This study was conducted in standard private veterinary practices that do not maintain institutional IACUCs. All dogs were clinical patients undergoing procedures indicated as part of their veterinary care, and study participation did not alter their surgical, anesthetic, or analgesic management. The study was conducted in accordance with American Animal Hospital Association (AAHA) Guidelines for Dental Care and Ethics [19]. Written informed owner consent was obtained prior to study enrollment (Supplementary Figure S1).

### 2.8. Laboratory Methods

1. Sample Information

In total, 25 canine plasma samples were used for vitamin and vitamin-like compound quantification analysis by Ultra-Performance Liquid Chromatography–Tandem Mass Spectrometry (UPLC-MS/MS).

2. Sample Preparation and LC-MS Analysis

Water-soluble vitamin assay:

In total, 50 μL of each plasma sample was mixed with 200 μL of an internal standard (IS) solution dissolved in 90% acetonitrile. The samples were vortexed at 3000 rpm for 1 min, followed by ultra-sonication in an ice-water bath for 2 min. After centrifugal clarification at 21,000 *g* for 10 min, the clear supernatants were collected and diluted fivefold with water. Nine serially diluted calibration solutions of water-soluble vitamins were prepared in the solvent- and concentration-matched IS solution. The concentration range of each compound was 0.000002 to 100 μM. Method 1: 10 μL aliquots of each sample solution or each calibration solution were injected to run UPLC-MRM/MS on an Agilent 1290 UHPLC system(Agilent Technologies, Inc., Santa Clara, CA, USA) coupled to an Agilent 6495C QQQ mass spectrometer (Agilent Technologies, Inc., Santa Clara, CA, USA) equipped with an electrospray ionization (ESI) source and operated in the positive ion mode. A C18 column (2.1 × 100 mm, 1.8 μm) and an ammonium acetate solution (A) and methanol (B) were used for binary solvent gradient elution under optimized conditions for the LC separation and MRM/MS detection. Method 2: 10 μL aliquots of each sample solution or each calibration solution were injected to run UPLC-MRM/MS on an Agilent 1290 UHPLC system coupled to a Sciex QTRAP 6500 Plus mass spectrometer (AB Sciex LLC, Framingham, MA, USA) equipped with an electrospray ionization (ESI) source and operated in the negative ion mode. A HILIC column (2.1 × 100 mm, 1.7 μm), an ammonium acetate solution (A) and acetonitrile (B) were used for binary solvent gradient elution under optimized conditions for the LC separation and MRM/MS detection.

Fat-soluble vitamin assay:

In total, 50 μL of each plasma sample was mixed with 250 μL of water, 50 μL of an internal standard solution of isotope-labeled vitamin D2, vitamin D3, retinal, and a-tocopherol and 450 μL of MTBE. The samples were vortexed for 1 min, followed by centrifugation at 21,000 *g* for 10 min. The clear supernatants were collected into 3 mL glass test tubes. The samples were extracted two more times again with MTBE, at 0.5 mL each time. After extraction and centrifugation, the clear supernatants from each sample were pooled and then dried under a nitrogen gas flow. The residues were dissolved in 0.1 mL of ethanol. Serially diluted, 9 calibration solutions of fat-soluble vitamins were prepared in the IS solution. The concentration range of each compound was 0.000002 to 0.5 μg/mL. In total, 10 μL aliquots of each sample solution or each calibration solution were injected to run UPLCMRM/MS on an Agilent 1290 UHPLC system coupled to a Sciex 7500 QQQ mass spectrometer (AB Sciex LLC, Framingham, MA, USA), which was equipped with an electrospray ionization source and operated in the positive ion mode. A 5 cm long C8 UPLC column and 0.1% formic acid in water (A) and acetonitrile (B) were used for binary solvent gradient elution under optimized conditions for the LC separation and MRM/MS detection.

### 2.9. Statistical Analysis Method

As this was an exploratory pilot study, no formal a priori sample-size calculation was performed. A sensitivity analysis based on the achieved group sizes (8 controls and 17 dogs with Stage 4 periodontal disease) indicated that, for a conventional two-sided independent-group comparison at α = 0.05 and 80% power, the minimum detectable standardized effect size was approximately Cohen’s d = 1.25. This calculation provides an approximate indication of the magnitude of effect detectable with the achieved sample sizes and does not account for the subsequent adjustment for multiple comparisons.

All analyses were performed using SAS 9.4 (SAS Institute Inc., Cary, NC, USA). A significance level of 0.05 was used. Normality was assessed using QQ plots, histograms, and skewness. Age was compared between groups with a Welch’s *t*-test and body weight with a Mann–Whitney test. Vitamin levels were log-transformed prior to analysis to remove skewness and approximate normality and are summarized as geometric means and medians (IQR). Of the 46 vitamins measured, nine were excluded from all statistical analyses as their values were below the limit of quantification (LOQ). Log-transformed vitamin levels were compared between groups (control vs. diseased), supplement use (yes vs. no), medication use (yes vs. no), and presence of underlying health conditions (yes vs. no) with Welch’s *t*-tests. When any values were below the LOQ, censored regression (Tobit) models were implemented using PROC QLIM with the LOQ as the lower censoring bound.

Associations between log-transformed vitamin levels and age (per 1 year), body weight (per 5 kg), and percent of teeth affected by Stage 4 periodontal disease (PD4) during the current procedure (per 10% increase in the teeth affected) were evaluated using linear regression when all values were above the LOQ, and censored regression (Tobit) via PROC QLIM when any values were below the LOQ. Regression coefficients were back-transformed to percent change using (e^(sβ) − 1) × 100, where s is the scaling factor, for ease of interpretation. *p*-values were adjusted for multiple comparisons using the adaptive linear step-up procedure of Benjamini and Hochberg (2000) [20]. Group comparisons were adjusted as a single family of 37 tests because they were the primary predictors. All remaining comparisons (supplements, medications, underlying conditions, age, body weight, and percent PD4) were adjusted together as a second family of 222 tests (37 vitamins × 6 predictors). Unadjusted *p*-values are also reported.

## 3. Analytical Results

### 3.1. Results

A total of 25 dogs were included in the analysis, comprising eight control dogs without periodontal disease and 17 dogs with severe periodontal disease (PD4). The mean ± SD age was 6.4 ± 5.4 years in the control group and 9.0 ± 3.0 years in the PD4 group (*p* = 0.24). Median (IQR) body weight was 18.6 (3.6–33.8) kg in the control group and 7.3 (3.5–21.8) kg in the PD4 group (*p* = 0.32).

#### 3.1.1. Periodontal Disease Status and Vitamin Concentrations

Following log transformation and adjustment for multiple comparisons, only vitamin B9 (folate) remained significantly associated with severe PD, with geometric mean concentrations approximately 28% lower in dogs with PD than in controls (0.00139 vs. 0.00193 μmol/L; adjusted *p* = 0.006).

In exploratory unadjusted analyses, several additional metabolites demonstrated lower geometric mean concentrations in dogs with PD, including vitamin D3 (*p* = 0.006), lipoic acid (*p* = 0.04), vitamin B6 pyridoxal phosphate (PLP) (*p* = 0.02), vitamin B6 pyridoxamine (*p* = 0.03), all-trans retinal (*p* = 0.008), γ-tocopherol (*p* = 0.04) and β-tocotrienol (*p* = 0.01). Lower concentrations of γ-tocotrienol (*p* = 0.05) and α-tocotrienol (*p* = 0.06) were also observed, although these differences were not statistically significant. Conversely, vitamin C (ascorbic acid) concentrations were higher in dogs with PD compared with controls (*p* = 0.0498).

#### 3.1.2. Periodontal Disease Severity and Vitamin Concentrations

Increasing PD severity (reported as % change per 10% increase in teeth affected by PD4) was associated with lower concentrations of several metabolites on unadjusted analyses, including lipoic acid (−4.9%; 95% CI, −9.3% to −0.3%; *p* = 0.049), vitamin B9 (folate) (−3.9%; 95% CI, −7.1% to −0.7%; *p* = 0.03), all-trans retinal (−21.7%; 95% CI, −32.4% to −9.4%; *p* = 0.003), and β-tocotrienol (−20.4%; 95% CI, −34.3% to −3.6%; *p* = 0.03). In contrast, PD severity was associated with higher concentrations of vitamin B2 flavin adenine dinucleotide (FAD) (+5.1%; 95% CI, 0.8% to 9.5%; *p* = 0.03), adenosylcobalamin (Ado-CBM) (+10.2%; 95% CI, 2.6% to 18.3%; *p* = 0.01), and methylcobalamin (Methyl-CBM) (+13.9%; 95% CI, 2.3% to 26.8%; *p* = 0.03). None of these associations remained significant after adjustment for multiple comparisons.

#### 3.1.3. Age and Vitamin Concentrations

Independent of PD, increasing age was associated with lower vitamin B1 (thiamine pyrophosphate) concentrations, which decreased by 20.3% per year (95% CI, −28.7% to −11.0%; adjusted *p* = 0.01). In unadjusted analyses, increasing age was also associated with lower concentrations of cyanocobalamin (Cyano-CBM), hydroxocobalamin (OH-CBM), and total vitamin B12, which decreased by 6.7% (95% CI, −11.4% to −1.8%; 2*p* = 0.01), 4.1% (95% CI, −7.1% to −1.1%; *p* = 0.01), and 4.4% (95% CI, −7.3% to −1.4%; *p* = 0.01) per year, respectively (adjusted *p* = 0.41 for all).

#### 3.1.4. Concurrent Medications, Supplements and Comorbidities

Twenty percent of dogs (5/25) were receiving glucosamine/chondroitin sulfate supplementation. Long-term medications were administered to 12% (3/25) of dogs and included oclacitinib (1/25), pimobendan (1/25), and fluoxetine (1/25). Pre-surgical anxiolytic medications were administered to 48% (12/25) of dogs and included trazodone plus gabapentin (5/25), trazodone alone (4/25), gabapentin alone (2/25), and a combination of fluoxetine, acepromazine, and gabapentin (1/25). Monthly antiparasitic preventatives were administered to 92% (23/25) of dogs, including flea, tick, and heartworm combination products (21/25) and heartworm-only preventatives (2/25). Twenty-eight percent (7/25) of dogs had documented comorbidities, including stable chronic valvular heart disease (2/25), a history of surgically corrected ectopic ureters (1/25), elevated liver enzymes (1/25), atopic dermatitis (1/25), recurrent urinary tract infections (1/25), and intervertebral disk disease (1/25). No significant associations were identified between vitamin concentrations and glucosamine/chondroitin supplementation, concurrent medication use, parasiticide use, or the presence of systemic comorbidities after adjustment for multiple comparisons.

## 4. Discussion

This small cross-sectional pilot study identified that vitamin B9 was significantly associated with severe periodontitis in dogs. Conclusions regarding alterations in other vitamin-related pathways in canine PD cannot be made at this time. This study may provide a foundation for future investigation.

In people, the following vitamins have been reported to be associated with periodontitis: vitamins C, D, A, B-complex, E [1,3,5,6,7,9,10,12] and vitamin K [8]. The mechanisms linking vitamin status and human periodontitis, though not fully understood, may provide a model for understanding vitamin aberrations in dogs. In people, vitamin deficits may contribute to dysregulated inflammatory responses [9]. In addition, the antioxidant properties of certain vitamins may support periodontal health [12]. Reduced circulating vitamin concentrations may reflect increased utilization of antioxidant and immunomodulatory nutrients in response to the oxidative stress associated with PD [21].

The current understanding of the relationship between vitamin levels and PD in veterinary patients remains limited. An interesting study that evaluated serum vitamin C and calcidiol (25-hydroxyvitamin D) in dogs with different grades of periodontitis revealed that both vitamins declined with increasing periodontitis severity, and that the decline was steeper for calcidiol [17]. Their results showed that lower serum concentrations were also associated with higher gingivitis and plaque indices and increasing age. These findings suggest potential associations between vitamin status and PD in dogs. Another recent study [16] evaluated folic acid in dogs with PD and demonstrated inhibition of *Porphyromonas gulae* growth and inflammatory cytokine production in vitro. In dogs receiving folic acid-containing dental chews for 28 days, *P. gulae* activity and halitosis were reduced, although plaque, calculus, and gingivitis scores were not significantly altered. Methodological differences in PD assessment, breed, genetics, age, sex and neutering status should be considered when comparing the findings of different studies. Established reference intervals for many vitamin concentrations in healthy dogs remain limited. Furthermore, differences in sample type, handling, and analytical methodology complicate direct comparison with previously reported concentrations, as reference intervals are method-dependent and values generated using different analytical platforms may not be directly comparable [22].

In our study, lower folate levels were found in dogs with periodontitis. Vitamin B refers to a group of eight essential water-soluble vitamins (B1—thiamine, B2—riboflavin, B3—niacin, B5—pantothenic acid, B6—pyridoxine, B7—biotin, B9—folate and B12—cobalamin) that play crucial roles in metabolism, energy production and various physiological functions [12,23,24,25]. While not all B vitamins have the same impact on periodontal health, several of them have been reported to be associated with PD in people, including vitamins B6, B9 and B12 [23]. Folate (vitamin B9) plays essential roles in DNA synthesis and repair, red blood cell formation, and one-carbon metabolism, which is critical for cell division and growth [23,24,25]. In people, deficiency in folate in particular is associated with increased severity of PD and worse outcomes [9,12,23,26,27,28]. The lower folate concentrations observed in dogs with severe periodontitis are also of interest given the findings of Shirahata et al. [16], who demonstrated antimicrobial and anti-inflammatory effects of folic acid in a canine periodontal disease model. However, their study evaluated folic acid administration rather than systemic folate status, and, therefore, the relationship between circulating folate concentrations and these effects remains unclear. Additionally, our study showed that in exploratory analyses, lower vitamin B9 (folate) concentrations were observed with increasing PD severity, with concentrations decreasing by 3.9% for every 10% increase in the proportion of teeth affected by PD4, although this association did not remain significant after adjustment for multiple comparisons. The relationship between B-vitamin status and canine PD remains incompletely understood and warrants further investigation.

Exploratory unadjusted analyses also identified lower concentrations of several vitamin-related metabolites in dogs with PD, including those related to vitamins D (vitamin D3), B6 (PLP and pyridoxamine), A (all-trans retinal), and E (γ-tocopherol and β-tocotrienol), as well as lipoic acid. Although these findings did not remain significant after adjustment for multiple comparisons, they may warrant further investigation in larger studies.

Associations between vitamin concentrations and age were independent of PD status. Even though this was a small study, increasing age was significantly associated with lower vitamin B1-thiamine pyrophosphate concentrations, with an estimated decline of 20.3% per year. Similar age-related decreases were observed for several vitamin B12 metabolites and total B12. Therefore, age should be considered when interpreting circulating vitamin concentrations in future canine nutritional and metabolomic studies.

Most human studies have evaluated the relationship between PD and either dietary vitamin intake or circulating vitamin concentrations, while a smaller body of work has examined the effects of vitamin supplementation on periodontal outcomes. The evidence is still lacking to support universal recommendations about daily vitamin intakes pertaining to oral health benefits in people [29]. It is plausible that targeted vitamin supplementation, in conjunction with routine periodontal preventive strategies such as tooth brushing and professional dental prophylaxis, may support periodontal health maintenance in dogs. Future controlled interventional longitudinal studies are needed for clinical translation. Client education on the importance of a complete and balanced diet for their pets is a practical recommendation.

This exploratory, cross-sectional pilot study investigating serum plasma vitamin levels in dogs with severe PD has various limitations. First, the cross-sectional design limits causal inferences. Second, the relatively small sample size and heterogeneous control group may have limited statistical power and impacted our results. Although age and body weight did not differ significantly between groups, this should not be interpreted as evidence of equivalence, and residual confounding by these factors cannot be excluded. As the study was conducted in a real-world clinical population, dietary intake and lifestyle factors were not standardized. Although dogs receiving nutritional supplements were excluded, variation in dietary vitamin content and bioavailability may have influenced plasma vitamin concentrations and represents a potential confounding factor. Methodologically, plasma was used for the measurement of both water- and fat-soluble vitamins. While serum is often preferred for the assessment of fat-soluble vitamins because of its reduced anticoagulant interference and the availability of established reference ranges [30], both serum and plasma are used in vitamin research. As such, differences in sample matrices may represent a source of variability when comparing vitamin concentrations across studies. Interpretation is further limited by the lack of established canine reference intervals for many vitamins and the method-dependent nature of reported concentrations. Further, an important limitation of the present study was that 25-hydroxyvitamin D [25(OH)D; calcidiol], the commonly used biomarker of vitamin D status, was not included in the available analytical panel. Consequently, the measured vitamin D analytes should not be interpreted as reflecting overall vitamin D status. Future studies incorporating calcidiol are warranted to specifically investigate the relationship between vitamin D status and canine periodontal disease. Lastly, the current study only demonstrates associations and cannot determine whether vitamin deficiency contributes to the development of PD or whether periodontal disease leads to decreased vitamin levels.

Future research in veterinary oral medicine should prioritize integrated approaches to better characterize the biological mechanisms underlying periodontal disease. Larger vitamin studies with balanced cohorts are needed to validate these findings and further characterize the associations between vitamin status and PD. Comparative studies of the oral microbiome, cytokine profiles, and metabolomic signatures across species may additionally provide deeper insights into PD pathogenesis.

## 5. Conclusions

Lower plasma folate concentrations were associated with severe periodontitis in dogs. Additional exploratory associations were identified for several vitamin metabolites but require validation in larger studies. Differences between canine and human vitamin profiles may indicate species-specific relationships between micronutrient status and PD. These findings support further investigation of vitamin status in canine PD, with further studies needed to determine their clinical and biological significance.

## Figures and Tables

**Table 1:** Patient signalment.

Patient	Control/PD4	Age	Sex	Breed	Body Weight (kg)	Body Weight: Small, Medium, Large
1	Control	6 years	Male neutered	Greyhound	33	Large
2	Control	9 months	Female entire	Toy Fox Terrier	2.7	Small
3	Control	1.5 years	Female spayed	Samoyed	18.1	Medium
4	Control	6 years	Male neutered	Labrador Retriever	34.5	Large
5	Control	15 years	Female spayed	Maltese	3.3	Small
6	Control	14 years	Female spayed	Mixed Breed	19	Medium
7	Control	5 years	Male neutered	Mastiff Cross	60.3	Large
8	Control	3 years	Male neutered	Pomeranian	3.8	Small
9	PD4	7 years	Male neutered	Chihuahua	2.5	Small
10	PD4	7 years	Male neutered	Beagle Mix	18.6	Medium
11	PD4	12 years	Male neutered	Siberian Husky	23.1	Medium
12	PD4	12 years	Female spayed	Toy Poodle Mix	3.5	Small
13	PD4	11 years	Male neutered	English Setter	29	Large
14	PD4	12 years	Male neutered	Pitbull Cross	31.3	Large
15	PD4	3 years	Female spayed	Miniature Daschund	4.4	Small
16	PD4	14 years	Female spayed	Yorkshire Terrier	2.2	Small
17	PD4	9 years	Male entire	Collie Cross	25	Medium
18	PD4	10 years	Female spayed	Papillon	4.8	Small
19	PD4	6 years	Female spayed	Pomeranian	2.8	Small
20	PD4	10 years	Male neutered	Beagle Mix	15.9	Medium
21	PD4	5 years	Female spayed	American Eskimo Dog	7.3	Small
22	PD4	10 years	Male neutered	Standard Daschund	6.8	Small
23	PD4	7 years	Female spayed	Cavalier King Charles Spaniel	21.8	Medium
24	PD4	6 years	Female spayed	Maltese × Poodle	1.9	Small
25	PD4	11 years	Female spayed	Pomeranian	10.9	Medium

Small = 0–10 kg; medium = 10–25 kg; large = 25+ kg.

**Table 2:** Stage of periodontal disease per American Veterinary Dental College (AVDC).

Stage of Periodontal Disease per American Veterinary Dental College (AVDC)	Description
Stage 0 (PD0)	Clinically normal; gingival inflammation or periodontitis is not clinically evident.
Stage 1 (PD1)	Gingivitis only without attachment loss; the height and architecture of the alveolar margin are normal.
Stage 2 (PD2)	Early periodontitis; less than 25% of attachment loss or, at most, there is a stage 1 furcation involvement in multirooted teeth. There are early radiologic signs of periodontitis. The loss of periodontal attachment is less than 25% as measured either by probing of the clinical attachment level or radiographic determination of the distance of the alveolar margin from the cementoenamel junction relative to the length of the root.
Stage 3 (PD3)	Moderate periodontitis—25–50% of attachment loss as measured either by probing of the clinical attachment level or radiographic determination of the distance of the alveolar margin from the cementoenamel junction relative to the length of the root, or there is a stage 2 furcation involvement in multirooted teeth.
Stage 4 (PD4)	Advanced periodontitis; more than 50% of attachment loss as measured either by probing of the clinical attachment level or radiographic determination of the distance of the alveolar margin from the cementoenamel junction relative to the length of the root, or there is a stage 3 furcation involvement in multirooted teeth.

Note: The degree of severity of periodontal disease (PD) relates to a single tooth; a patient may have teeth that have different stages of PD.

**Table 3:** Clinical diagnosis and treatment.

Patient	Control/PD4	Diagnosis	Teeth with PD4 (% of Teeth Present)	Treatment
1	Control	PD1	0	COHAT
2	Control	PD0 Class 1 Malocclusion—Linguoversion of mandibular right canine tooth	0	COHAT Vital pulp therapy of mandibular right canine tooth Odontoplasty mandibular incisors
3	Control	PD0 Uncomplicated crown fracture of maxillary right canine tooth	0	COHAT Restoration of maxillary right canine tooth
4	Control	PD0 Class 1 Malocclusion—Mild linguoversion of mandibular left canine tooth and mandibular right canine tooth	0	COHAT Gingivectomy at the interdental space between maxillary right third incisor tooth and maxillary right canine tooth, and maxillary left third incisor tooth & maxillary left canine tooth
5	Control	PD1	0	COHAT
6	Control	PD1	0	COHAT
7	Control	PD1 Complicated crown fracture of maxillary right canine tooth and mandibular left first molar tooth Uncomplicated crown fracture of maxillary right fourth premolar tooth	0	COHAT Standard root canal therapy at maxillary right canine tooth. Odontoplasty at maxillary left fourth premolar tooth. Extraction of a mandibular left first molar tooth.
8	Control	PD1	0	COHAT
9	PD4	PD4—3 teeth	27	COHAT Extraction of all PD4 teeth
10	PD4	PD4—12 teeth	34	COHAT Extraction of all PD4 teeth
11	PD4	PD4—7 teeth Gingival Enlargement—generalized	18	COHAT Extraction of all PD4 teeth Gingivectomy at multiple teeth
12	PD4	PD4—5 teeth	33	COHAT Extraction of all PD4 teeth
13	PD4	PD4—18 teeth	53	COHAT Extraction of all PD4 teeth
14	PD4	PD4—6 teeth	19	COHAT Extraction of all PD4 teeth
15	PD4	PD4—14 teeth	33	COHAT Extraction of all PD4 teeth
16	PD4	PD4—7 teeth	21	COHAT Extraction of all PD4 teeth
17	PD4	PD4—11 teeth	42	COHAT Extraction of all PD4 teeth
18	PD4	PD4—13 teeth	68	COHAT Extraction of all PD4 teeth
19	PD4	PD4—18 teeth	69	COHAT Extraction of all PD4 teeth
20	PD4	PD4—9 teeth	33	COHAT Extraction of all PD4 teeth
21	PD4	PD4—23 teeth	59	COHAT Extraction of all PD4 teeth
22	PD4	PD4—3 teeth	10	COHAT Extraction of all PD4 teeth
23	PD4	PD4—25 teeth	60	COHAT Extraction of all PD4 teeth
24	PD4	PD4—21 teeth	72	COHAT Extraction of all PD4 teeth
25	PD4	PD4—21 teeth	50	COHAT Extraction of all PD4 teeth

Abbreviations: PD0 = Clinically normal (PD0). PD1 = Periodontal disease, gingivitis only (without attachment loss) (PD1). PD4 = Advanced periodontitis (>50% attachment loss) (PD4). COHAT = Comprehensive Oral Health Assessment and Treatment (involves cleaning, polishing, probing, full mouth dental radiographs). PD4 percentage was calculated as the number of teeth classified as PD4 divided by the total number of teeth present at the time of examination × 100.

## Data Availability

The data supporting the findings of this study are available from the corresponding authors upon reasonable request.

## Supplementary Materials

The following supporting information can be downloaded at: https://www.mdpi.com/article/10.3390/vetsci13090916/s1, Table S1: **Vitamin levels by Group (Control vs. Diseased) (μmol/L)**; Figure S1: **Informed Client Consent Forms**.

## Abbreviations

The following abbreviations are used in this manuscript:

PD	Periodontal Disease
CBC	Complete Blood Count
PD0	Clinically normal
PD1	Periodontal disease, gingivitis only (without attachment loss)
PD4	Advanced periodontitis (>50% attachment loss)
COHAT	Comprehensive Oral Health Assessment and Treatment (involves cleaning, polishing, probing, full mouth dental radiographs)
