# Supplementary material for: Vitamins and Vitamin-like Compounds in Severe Canine Periodontal Disease: A Prospective Exploratory Cross-Sectional Pilot Study
Source: Vet Sci. 2026 Sep 7;13(9):916. doi: 10.3390/vetsci13090916 (PMC13611851; doi:10.3390/vetsci13090916)
Supplement: Supplementary file 1 [file vetsci-13-00916-s001.zip › vetsci-4435099-supplementary.pdf]

Table S1. Vitamin levels by Group (Control vs. Diseased) (μmol/L)

Vitamin	Level	Geometric Mean	Median (Q1, Q3)	(Min–Max)	Unadj. p	Adj. p
4-Aminobenzoic acid	Control	0.174	0.343 (0.231, 0.424)	(<0.0012 – 0.674)	0.05	0.13
	Diseased	0.019	0.062 (<0.0012, 0.231)	(<0.0012 – 0.438)		
Lipoic acid	Control	0.00409	0.00425 (0.00334, 0.00489)	(0.00300 – 0.00548)	0.04	0.13
	Diseased	0.00321	0.00281 (0.00264, 0.00367)	(0.00183 – 0.00681)		
Vitamin B1-thiamine	Control	0.038	0.039 (0.023, 0.077)	(0.011 – 0.114)	0.20	0.33
	Diseased	0.025	0.023 (0.019, 0.030)	(0.013 – 0.085)		
Vitamin B12	Control	<0.0016	<0.0016 (<0.0016, <0.0016)	(<0.0016 – <0.0016	a	a
	Diseased	0.00168	<0.0016 (<0.0016, <0.0016)	(<0.0016 – 0.00267)		
Vitamin B1-Thiamine-P	Control	0.0033	<0.0016 (<0.0016, 0.0124)	(<0.0016 – 0.0379)	a	a
	Diseased	0.0030	<0.0016 (<0.0016, <0.0016)	(<0.0016 – 0.0789)		
Vitamin B1-Thiamine-PP	Control	0.0080	0.0136 (<0.0016, 0.0251)	(<0.0016 – 0.0357)	0.72	0.72
	Diseased	0.0085	0.0139 (0.0051, 0.0192)	(<0.0016 – 0.0307)		
Vitamin B2	Control	0.067	0.067 (0.062, 0.077)	(0.044 – 0.091)	0.48	0.50
	Diseased	0.072	0.074 (0.065, 0.088)	(0.042 – 0.108)		
Vitamin B2_FAD	Control	1.39	1.34 (1.18, 1.63)	(1.16 – 1.82)	0.14	0.29
	Diseased	1.62	1.65 (1.27, 1.94)	(0.90 – 2.88)		
Vitamin B2-Riboflavin-5 P(FMN)	Control	0.00169	<0.0016 (<0.0016, <0.0016)	(<0.0016 – 0.00253)	a	a
	Diseased	0.00172	<0.0016 (<0.0016, <0.0016)	(<0.0016 – 0.00552)		
Vitamin B3-Nicotinic acid	Control	0.0223	0.0206 (0.0156, 0.0339)	(0.0124 – 0.0435)	0.29	0.39
	Diseased	0.0184	0.0181 (0.0139, 0.0231)	(0.0108 – 0.0302)		
Vitamin B3-Nicotinamide	Control	1.99	1.51 (0.92, 4.77)	(0.86 – 7.39)	0.20	0.33

Vitamin	Level	Geometric Mean	Median (Q1, Q3)	(Min–Max)	Unadj. p	Adj. p
	Diseased	1.26	1.32 (0.97, 1.81)	(0.71 – 2.84)		
Vitamin B5-Pantothenate	Control	0.24	0.19 (0.14, 0.39)	(0.11 – 1.11)	0.32	0.39
	Diseased	0.18	0.18 (0.15, 0.21)	(0.08 – 0.34)		
Vitamin B6-Pyridoxal	Control	0.066	0.068 (0.053, 0.082)	(0.032 – 0.143)	0.45	0.48
	Diseased	0.055	0.062 (0.047, 0.081)	(0.011 – 0.146)		
Vitamin B6-Pyridoxal-P	Control	0.0319	0.0341 (0.0244, 0.0448)	(0.0150 – 0.0558)	0.02	0.09
	Diseased	0.0179	0.0203 (0.0136, 0.0251)	(0.0055 – 0.0467)		
Vitamin B6-Pyridoxamine	Control	0.0064	0.0065 (0.0054, 0.0075)	(0.0040 – 0.0104)	0.03	0.12
	Diseased	0.0040	0.0044 (0.0022, 0.0057)	(0.0019 – 0.0254)		
Vitamin B6-Pyridoxine	Control	0.00066	0.00059 (0.00052, 0.00086)	(0.00041 – 0.00117)	0.97	0.97
	Diseased	0.00066	0.00064 (0.00057, 0.00072)	(0.00049 – 0.00100)		
Vitamin B7-Biotin	Control	0.0044	0.0046 (0.0036, 0.0049)	(0.0029 – 0.0077)	0.65	0.65
	Diseased	0.0041	0.0043 (0.0025, 0.0066)	(0.0015 – 0.0106)		
Vitmin B9-5MTHF	Control	<0.0006	<0.0006 (<0.0006, <0.0006)	(<0.0006 – <0.0006)	a	a
	Diseased	<0.0006	<0.0006 (<0.0006, <0.0006)	(<0.0006 – <0.0006)		
Vitmin B9-Folate	Control	0.00193	0.00195 (0.00173, 0.00210)	(0.00156 – 0.00244)	<.001	0.006
	Diseased	0.00139	0.00139 (0.00119, 0.00156)	(0.00095 – 0.00187)		
Vitamin C_Ascorbic acid	Control	0.00131	0.00129 (0.00123, 0.00140)	(0.00116 – 0.00147)	0.05	0.13
	Diseased	0.00149	0.00141 (0.00134, 0.00157)	(0.00120 – 0.00328)		
all-trans retinol	Control	0.91	1.73 (0.63, 5.22)	(0.00 – 6.50)	0.97	0.97
	Diseased	0.95	1.14 (0.89, 2.25)	(0.00 – 4.05)		
all-trans retinal	Control	0.0110	0.0118 (0.0051, 0.0219)	(0.0029 – 0.0456)	0.008	0.07

Vitamin	Level	Geometric Mean	Median (Q1, Q3)	(Min–Max)	Unadj. p	Adj. p
	Diseased	0.0031	0.0025 (0.0016, 0.0038)	(0.0009 – 0.0465)		
all-trans retinoic acid	Control	0.0043	0.0043 (0.0027, 0.0063)	(0.0026 – 0.0082)	0.32	0.39
	Diseased	0.0034	0.0030 (0.0023, 0.0050)	(0.0015 – 0.0164)		
Retinyl acetate	Control	<0.000006	<0.000006 (<0.000006, <0.000006)	(<0.000006 – <0.000006)	a	a
	Diseased	<0.000006	<0.000006 (<0.000006, <0.000006)	(<0.000006 – <0.000006)		
Retinyl palmitate	Control	0.37	0.43 (0.20, 0.63)	(0.10 – 1.33)	0.67	0.67
	Diseased	0.32	0.37 (0.26, 0.48)	(0.03 – 1.42)		
1-OH vitamin D2	Control	<0.000006	<0.000006 (<0.000006, <0.000006)	(<0.000006 – <0.000006)	a	a
	Diseased	<0.000006	<0.000006 (<0.000006, <0.000006)	(<0.000006 – <0.000006)		
Vitamin D2	Control	0.00017	0.00016 (0.00012, 0.00028)	(0.00006 – 0.00047)	0.53	0.53
	Diseased	0.00021	0.00019 (0.00015, 0.00027)	(0.00009 – 0.00130)		
1-OH vitamin D3	Control	<0.000006	<0.000006 (<0.000006, <0.000006)	(<0.000006 – <0.000006)	a	a
	Diseased	<0.000006	<0.000006 (<0.000006, <0.000006)	(<0.000006 – <0.000006)		
Vitamin D3	Control	0.00031	0.00034 (0.00023, 0.00044)	(0.00012 – 0.00057)	0.006	0.07
	Diseased	0.00012	0.00010 (0.00007, 0.00020)	(0.00001 – 0.00144)		
α-tocopherol	Control	1.37	3.07 (1.33, 4.74)	(0.01 – 5.59)	0.83	0.83
	Diseased	1.66	2.01 (1.68, 2.79)	(0.00 – 6.01)		
β-tocopherol	Control	0.093	0.127 (0.039, 0.186)	(0.022 – 0.349)	0.16	0.31
	Diseased	0.050	0.047 (0.028, 0.111)	(0.008 – 0.209)		
γ-tocopherol	Control	0.00019	0.00022 (0.00016, 0.00032)	(0.00005 – 0.00040)	0.04	0.13
	Diseased	0.00006	0.00005 (0.00002, 0.00013)	(<0.000013 – 0.00254)		

Vitamin	Level	Geometric Mean	Median (Q1, Q3)	(Min–Max)	Unadj. p	Adj. p
α-tocotrienol	Control	0.0077	0.0121 (0.0064, 0.0160)	(0.0004 – 0.0266)	0.06	0.14
	Diseased	0.0025	0.0029 (0.0020, 0.0046)	(<0.000013 – 0.0111)		
β-tocotrienol	Control	0.0033	0.0048 (0.0014, 0.0082)	(0.0004 – 0.0106)	0.010	0.07
	Diseased	0.0008	0.0009 (0.0003, 0.0018)	(0.0001 – 0.0050)		
γ-tocotrienol	Control	0.0054	0.0043 (0.0033, 0.0097)	(0.0028 – 0.0143)	0.05	0.13
	Diseased	0.0019	0.0027 (0.0017, 0.0032)	(<0.000013 – 0.0083)		
Tocopheryl acetate	Control	0.0014	0.0010 (0.0009, 0.0020)	(0.0006 – 0.0082)	0.12	0.26
	Diseased	0.0007	0.0005 (0.0004, 0.0008)	(0.0002 – 0.0193)		
Vitamin K1	Control	0.00102	0.00092 (0.00081, 0.00120)	(0.00071 – 0.00208)	0.39	0.44
	Diseased	0.00089	0.00087 (0.00072, 0.00103)	(0.00058 – 0.00227)		
Vitamin K2-MK-4	Control	0.000253	0.000279 (0.000142, 0.000369)	(0.000123 – 0.000656)	0.21	0.33
	Diseased	0.000179	0.000169 (0.000122, 0.000247)	(0.000037 – 0.000603)		
Vitamin K2-MK-7	Control	0.000170	0.000162 (0.000142, 0.000209)	(0.000120 – 0.000252)	0.22	0.33
	Diseased	0.000136	0.000147 (0.000097, 0.000221)	(0.000053 – 0.000525)		
Vitamin K2-MK-9	Control	<0.000032	<0.000032 (<0.000032, <0.000032)	(<0.000032 – <0.000032)	a	a
	Diseased	<0.000032	<0.000032 (<0.000032, <0.000032)	(<0.000032 – <0.000032)		
Vitamin K3	Control	0.000018	<0.000013 (<0.000013, 0.000032)	(<0.000013 – 0.000032)	a	a
	Diseased	<0.000013	<0.000013 (<0.000013, <0.000013)	(<0.000013 – <0.000013)		
Ado-CBM	Control	0.068	0.061 (0.049, 0.103)	(0.034 – 0.160)	0.39	0.44
	Diseased	0.083	0.073 (0.070, 0.109)	(0.037 – 0.291)		
Cyano-CBM	Control	0.095	0.088 (0.071, 0.116)	(0.051 – 0.257)	0.24	0.35

Vitamin	Level	Geometric Mean	Median (Q1, Q3)	(Min–Max)	Unadj. p	Adj. p
	Diseased	0.073	0.072 (0.057, 0.085)	(0.015 – 0.271)		
Methyl-CBM	Control	0.0057	0.0051 (0.0031, 0.0101)	(0.0023 – 0.0209)	0.30	0.39
	Diseased	0.0080	0.0094 (0.0075, 0.0109)	(0.0016 – 0.0344)		
OH-CBM	Control	0.70	0.68 (0.53, 0.83)	(0.45 – 1.46)	0.82	0.82
	Diseased	0.67	0.63 (0.55, 0.83)	(0.44 – 1.71)		
Total_B12	Control	0.88	0.85 (0.67, 1.11)	(0.56 – 1.71)	0.79	0.79
	Diseased	0.85	0.79 (0.67, 1.05)	(0.55 – 2.30)		

Sample sizes: Control: n=8, Diseased: n=17.

Values at or below the limit of quantification are shown as the LOQ value. Geometric mean, median, Q1, Q3, min, and max calculated on original scale. p-values from Satterthwaite t-test on log-transformed values (censored regression for vitamins with values below LOQ). Adj. p-value: Adaptive false discovery rate correction. Bold indicates $p < 0.05$.

a Not analyzed due to excessive values below the limit of quantification.

Supplementary Figure S1: **Informed Client Consent Forms**

INFORMED CONSENT FORM

CLINICAL STUDY:
Assessment of vitamins, the metabolome and key cytokines in canine patients with severe periodontitis versus periodontally healthy patients

Background Information

Periodontal disease is a progressive, chronic, inflammatory disease that when left untreated, can lead to destruction of tissue supporting teeth, and ultimately the loss of the tooth. Periodontal disease is one of the most common diseases diagnosed in generalist veterinary practices. While severity, progression and an underlying predisposition for periodontitis appears multifactorial, it is well established that periodontal disease in companion animals progresses with age, and that smaller breeds appear more susceptible.

In people, it is well described that vitamins play an important role in maintaining periodontal health and in preventing periodontal disease. Numerous studies in humans show that there appears to be an association between increased risk and/or severity of periodontal disease, with decreased levels of vitamin's A, B12, C, and D. Further studies are needed in the veterinary literature to understand this relationship further in our pets.

This study aims to evaluate if a comprehensive vitamin profile differs between patients affected by severe periodontal disease, versus a periodontially healthy cohort. This is clinically very important and relevant for multiple reasons: this may be invaluable as a prognostic indicator for periodontal disease, it may allow further investigation into vitamin affiliations with other oral inflammatory conditions affecting our companion animals, as well as allow for more possible preventative methods and treatment options in affected companion animals. It will also give us a better understanding to the multifaceted nature of periodontal disease pathogenesis and risks in our pets. The testing set also tests for vitamin E and vitamin K: we are interested to see if there are any difference in these results, however this is not the focus of the study.

The sample needed is one blood sample, which only needs to be taken once. Collection of samples will be achieved by routine blood collection. To minimize additional procedures, this will be performed at the same time as routine pre-anesthestic bloodwork. After collection, the blood samples are transported in a cooler and were stored at 4°C pending analysis.

Concurrently and with the same blood sample, we will evaluate the metabolome with untargeted mass chromatography and a cytokine profile in patients with severe periodontal disease versus healthy controls. Metabolomes are small molecule chemicals, or metabolites that are crucial in organisms growth, maintenance and normal function. Cytokines are a diverse group of small signaling proteins that play crucial roles within the immune system. Finally, noninvasive sterile swabs will be collected from the mouth of periodontitis patients and healthy controls to assess the micriobiome (bacteria). The data obtained from this study is for the benefit of the scientific community.

This study is being performed with patients being treated at the following institutions:

- Veterinary Dental Services LLC. Boxborough, MA.

This study is being investigated by primary investigators:

- Dr. Suzanna Hatunen, BVSc, Dipl.AVDC. Veterinary Dental Services LLC. Boxborough, MA.
- Dr. Jamie Anderson, DVM, MS, Dipl.AVDC, Dipl.ACVIM, Adjunct Professor School of Dental Medicine, University of Pennsylvania.

Samples will be evaluated by the reference laboratory **Creative Proteomics.**

(Place patient ID sticker here)

INFORMED CONSENT FORM

I hereby grant informed consent for inclusion of my dog in the following study:

Assessment of vitamins, the metabolome and key cytokines in canine patients with severe periodontitis versus periodontally healthy patients

1. I grant permission for the doctor and/or veterinary technician to collect a fasted blood sample from my dog (total needed 3mL); this will be taken during the same blood collection period as the required pre-anesthestic bloodwork. If pre-anesthesic bloodwork is not needed, then this blood sample would be a small additional process. I understand that the collection process is minimally-invasive with minimal risk. I understand blood collection is only needed one time. I understand that these samples are being analyzed for research purposes.

2. I grant permission for the doctor to attain 3 sterile cotton tip swabs from my dog, while they are under anesthesia to be used for micriobiome testing.

3. I understand that the blood sample + sterile swab samples may contribute to more than one published study, depending on the results attained.

4. I confirm that my pet is not on vitamin supplements, corticosteroids or antibiotics.

5. I agree to give permission for my pet's medical history to be used for the study; I acknowledge that **all data will be anonymized** and there will be **nothing published that will identify any one specific patient**.

6. I authorize obtaining photographs of my pets oral cavity, if necessary, and I give my permission to publish **anonymized data** obtained from this study for the benefit of the scientific community.

7. I understand this data is for research purposes, and I do not expect the results returned to me. The results of this study/studies will not alter the course of my dog's treatment.

A copy of this form, including Background Information, has been given to me. In addition, I have been given an opportunity to ask any questions I have concerning this study, and all such questions have been answered to my satisfaction.

____

Client's signature   Date

____

Clinician's signature   Date
